# Detection of Possible Resistance Mechanisms in Uropathogenic *Escherichia coli* Strains Isolated from Kidney Transplant Recipients Based on Whole Genome Sequencing

**DOI:** 10.3390/biom15020260

**Published:** 2025-02-11

**Authors:** Soraya Herrera-Espejo, Alejandro Rubio, Lucía Ceballos-Romero, Jerónimo Pachón, Elisa Cordero, Antonio J. Pérez-Pulido, María Eugenia Pachón-Ibáñez

**Affiliations:** 1 Clinical Unit of Infectious Diseases, Microbiology and Parasitology, Institute of Biomedicine of Seville (IBiS), Virgen del Rocio University Hospital/CSIC/University of Seville, 41013 Seville, Spain; sherrera-ibis@us.es (S.H.-E.); lceballos-ibis@us.es (L.C.-R.); mpachon-ibis@us.es (M.E.P.-I.); 2 Andalusian Centre for Developmental Biology (CABD, UPO-CSIC-JA), Faculty of Experimental Sciences (Genetics Area), University Pablo de Olavide, 41013 Seville, Spain; arubval@upo.es; 3 Institute of Biomedicine of Seville (IBiS), Virgen del Rocio University Hospital/CSIC/University of Seville, 41013 Seville, Spain; pachon@us.es; 4 Department of Medicine, School of Medicine, University of Seville, 41004 Seville, Spain; 5 CIBER de Enfermedades Infecciosas (CIBERINFEC), Instituto de Salud Carlos III, 28029 Madrid, Spain

**Keywords:** whole-genome sequencing, antimicrobial resistance, uropathogenic *Escherichia coli*

## Abstract

Background: Urinary tract infections are a global health concern, with uropathogenic *Escherichia coli* (UPEC) accounting for 80–90% of cases. Given the rise in antimicrobial resistance, our aim was to elucidate the genetic mechanisms behind low-level resistance to ciprofloxacin and fosfomycin (LLCR and LLFR) in UPEC strains, using whole-genome sequencing (WGS) to identify point mutations in chromosomal and plasmid genes. Methods: A cohort UPEC was collected from kidney transplant recipients at the Virgen del Rocío University Hospital, Spain. Minimum inhibitory concentrations were determined for ciprofloxacin and fosfomycin to categorize strains into LLCR and LLFR. Twenty strains were selected for WGS, with genome annotations. Point mutations were identified and analyzed using alignment tools, and protein stability changes were predicted. Results: LLCR strains exhibited mutations in key quinolone resistance-determining regions of the *gyrA* gene, in 83% of cases. The *qnrS1* plasmid gene was found in 17% of LLCR strains. LLFR strains showed mutations in the *glpT* and *cyaA* genes. Mutations in the *uhp* gene family were linked to the fosfomycin-resistant phenotype, suggesting a multi-step resistance evolution mechanism. Conclusions: This study highlights the complex interplay between chromosomal and plasmid genes in UPEC’s resistance to ciprofloxacin and fosfomycin. The findings contribute to understanding low-level resistance mechanisms and may guide the development of novel therapeutic strategies to combat multidrug-resistant strains.

## 1. Introduction

Urinary tract infections (UTIs) are a common health problem that affects a significant portion of the global population. Every year, around 150 million people worldwide develop UTIs, with substantial social and economic costs [1,2]. UTIs are more common in women, with an estimated 40% developing at least one UTI during their lifetime, and 11% experiencing an episode of UTI each year [2]. The mean cost of each UTI is estimated at EUR 5700 and EUR 6987 in Europe and USA, respectively [3,4]. UTIs are caused by the presence of bacteria (≥10^5^ CFU/mL) in the urine with or without symptoms associated (asymptomatic bacteriuria). Symptomatic UTIs are classified according to severity as urosepsis syndrome, pyelonephritis or upper UTI (kidney infection), and cystitis or lower UTI (bladder infection) [5]. Moreover, UTI is a common and serious problem among kidney transplant recipients (KTR), ranging from 40% to 50% [6,7]. This prevalence depends on factors such as preventive strategies and transplant characteristics. Several factors can increase the susceptibility to develop a UTI [8]. Among the risks that UTI might cause in KTR include immunosuppression, bladder catheterization, ureteral stents, deceased donor transplantation, and acute rejection episodes [9].

Uropathogenic *Escherichia coli* (UPEC) is the primary cause of UTIs acquired in the community, accounting for 80–90% of cases [1,2]. UPEC can be classified into different groups based on the presence of genomic pathogenicity islands (PAIs) and virulence factors [10]. UPEC uses a wide variety of virulence factors to colonize the bladder, including lipopolysaccharide (LPS), polysaccharide capsule, flagella, pili, and TonB-dependent iron-uptake receptors [8], all of which are potential targets for the development of new drugs and/or vaccines.

At present, despite the increase in antimicrobial resistance, there are numerous options for the treatment of UTIs. The European Antimicrobial Resistance Report 2021 (EARS-Net) informed that approximately 50% of *E. coli* strains were resistant to at least one group of antimicrobials. Ciprofloxacin resistance rates have increased up to 50% in the global scenario [11]. Specifically, in Spain, resistance rates of up to 30% for ciprofloxacin and 17% for aminoglycosides have been reported [12]. One of the primary mechanisms of antibiotic resistance in bacteria is the acquisition of genetic mutations that confer resistance to one or more antibiotics. Point mutations, which involve changes in a single nucleotide within the genome, are a common type of genetic mutation that can lead to antibiotic resistance [13]. Moreover, point mutations can be classified as synonymous (mutations or alterations that do not change the amino acid sequence of the protein) or non-synonymous (mutations that change the amino acid sequence of the protein, resulting in substitutions, deletions…), and occur spontaneously or can be induced by exposure to the antibiotics. These mutations can lead to changes in the target sites or efflux pumps of antibiotics, or alterations in the bacterial cell wall or membrane that reduce antibiotic uptake [14]. The study of antibiotic resistance in bacteria has traditionally relied on phenotypic methods, such as testing to determine the susceptibility/resistance profile of a given strain. However, with the arrival of next-generation sequencing technologies and bioinformatics tools, it is now possible to study the genetic basis of antibiotic resistance in more detail [15]. Currently, the most commonly used antibiotics are fosfomycin, ciprofloxacin, and amoxicillin-clavulanate for UTIs caused by UPEC [16]. Chromosomal and plasmid-mediated mechanisms on their own might confer low-level ciprofloxacin or fosfomycin resistance (LLCR or LLFR), which is clinically translated as a susceptible phenotype. However, the accumulation of multiple mutations could lead to clinical resistance [17]. These cumulative antimicrobial resistance genes could be acquired in nature from other bacteria [18] or even phages [19].

Thus, the aim of this study was to analyze chromosomal and plasmid genes involved in low-level resistance to ciprofloxacin and/or fosfomycin in UPEC strains collected from a well-characterized cohort of KTR, to identify point mutations that might be responsible for these. By characterizing these low-level genetic mechanisms of resistance, we seek to better understand the mechanisms underlying resistance of the mentioned antibiotics. Ultimately, the results may contribute to the development of new strategies to combat infections caused by these strains, for example, by identifying new therapeutic targets or by optimizing treatment.

## 2. Materials and Methods

### 2.1. Antimicrobial Susceptibility, Whole-Genome Sequencing and Multilocus Sequence Typing (MLST) of Escherichia coli Strains

One hundred and fifteen strains were collected in an observational cohort of adult KTRs with *E. coli* and UTI episodes (cystitis and asymptomatic bacteriuria [AB]), who signed the informed consent form (ethical approval numbers: FIS-CIP-2016-01 and FIS-FOS-2020-01) and attended as outpatients at the Virgen del Rocío University Hospital, Seville, Spain, from January 2017 to December 2019 [20]. Clinical strains were collected, and the hospital microbiology service identified the bacterial strains using a MicroScan WalkAway^®^ Plus system (Beckman Coulter, Nyon, Switzerland), and performed susceptibility testing with standard tests. Moreover, ciprofloxacin and fosfomycin Minimal Inhibitory Concentrations (MICs) were determined in duplicate, by broth microdilution or agar diffusion, respectively [20]. The results were interpreted according to the European Committee on Antimicrobial Susceptibility Testing (EUCAST) breakpoints for both antibiotics [21]. Antimicrobials to perform the MIC were purchased as standard powders (Sigma-Aldrich, Madrid, Spain) and *E. coli* ATCC 25922 was used as a quality control strain. MIC breakpoints used to designate susceptibility and resistance for ciprofloxacin were ≤0.001 µg/mL and >0.50 µg/mL and, in the case of fosfomycin, the breakpoint used to designate resistance was >8 µg/mL. MIC values of >0.06 to 0.5 mg/L and 4 to 8 mg/L were considered to be classified as LLCR and LLFR, respectively.

After MIC determinations, 20 UPEC strains were selected for in silico studies, 12 LLCR and 8 LLFR. Briefly, DNA from these strains was extracted (QIAamp^®^ DNA Mini Kit, Venlo, The Netherlands). Sequencing was performed using the MiSeq platform (Illumina), following standard protocols for WGS paired-end, producing 2 × 300 bp fragment reads. Unicycler was chosen for de novo assembly of the reads into contigs [22] (BioProject number: PRJNA1219036). Prodigal v2.6.3 was used to predict the protein-coding genes and translate them into their corresponding amino acid sequences [23]. The bacterial taxonomic division of the UniProt v2023_05 database was used for the functional annotation of the predicted proteins, using Sma3s v2 [24].

The contig files obtained from the assembly of each UPEC isolate were uploaded to the MLST web server (v. 2.0) of the Centre for Genomic Epidemiology (CGE) to perform MLST, following the schemes of Pasteur [25] and Achtman [26]. The settings for the analysis of these three web-based programs were established using a threshold of 90% identity and 80% for sequence coverage.

### 2.2. Quinolone and Fosfomycin Resistant Genes Sequence Alignments and Analysis

For the study of ciprofloxacin resistance genes, a total of eleven plasmid genes (*aac6’-1b-cr*, *qnrA*, *qnrB*, *qnrS*, *qnrC*, *qnrD*, *qnrE*, *qepA*, *oqxA*, *oqxB*, and *crpP*) and eight chromosomal genes (*gyrA*, *gyrB*, *parC*, *parE*, *marR*, *acrR*, *norB*, and *rpoB*) associated with quinolone resistance were examined. In the case of fosfomycin, eight plasmid genes (*fosA3*, *fosA4*, *fosA5*, *fosB*, *fosX*, *fosC*, *fomA*, and *fomB*) and nine chromosomal genes (*murA*, *uhpA*, *uhpB*, *uhpC*, *uhpT*, *glpT*, *ptsl*, *cyaA*, and *crp*) were analyzed. To examine these genes, the amino acid sequences of the encoding proteins predicted by Prodigal were aligned, using MAFFT (v. 7.526), to the reference amino acid sequences of the proteins obtained from The Comprehensive Antibiotic Resistance (CARD, v. 5.0.2) with default settings [27]. The pheatmap library from the R language was used to create a heatmap with the results obtained [28].

Subsequently, the structural stability of the mutated proteins was investigated using the Protein Variation Effect Analyzer (Provean v. 2.6.3) [29] and the CUPSAT [30] and DDMut [31] algorithms, which calculate Gibbs energy to predict changes in protein stability. To study the effect of mutations on the folding of the protein encoded by the *uhpB* gene, a 3D affine crystal structure was generated with the algorithm AlphaFold [32] and visualized using PyMol [33], representing the different mutations calculated in the previous steps of the study.

### 2.3. Identification of Unknown Resistance and Virulence Mechanisms

The data assembled from the 20 strains were used to identify resistance mechanisms. For this, CGE’s ResFinder web server (v. 4.1) [34,35], CGE’s Plasmid Finder web server (v. 2.1) [36,37], and NCBI’s AMRFinderPlus (v. 3.11) [38] were used for the acquired antimicrobial resistance genes. The thresholds for the analysis in these three web-based programs were established as 90% identity and 80% sequence coverage. CGE’s VirulenceFinder (v. 2.0.5) [35,39,40] web server was used for the identification of genes encoding virulence factors, with thresholds of 90% identity and 80% sequence coverage.

## 3. Results

### 3.1. Antimicrobial Susceptibility and Multilocus Sequence Typing (MLST) of Escherichia coli Strains Selected for Sequencing

MICs were determined for the 115 UPEC strains from the patients included in the cohort. Of these, 20 strains presented low-level resistance: 12 were categorized as LLCR and 8 as LLFR (Table 1 and Table 2). Demographics and characteristics of KTRs with UTI by LLCR or LLFR *E. coli* strains are described in Supplementary Table S1. LLCR strains exhibited a range of MIC values from 0.12 to 0.50 µg/mL and LLFR from 4 to 8 µg/mL. Two ciprofloxacin-susceptible strains (strains 5 and 44, MIC ≤ 0.015 mg/L) and two fosfomycin-susceptible strains (strains 145 and 139, MIC = 1 and 0.5 mg/L, respectively) were included as susceptibility controls.

Including the susceptibility controls, Achtman’s MLST scheme appeared to be more accurate assigning ST to 11/14 (78.6%) and 8/10 (80.0%) of the studied strains, for ciprofloxacin and fosfomycin, respectively. Independently of the scheme used, the strains presented a varied clonality without any single clone standing out (Table 1 and Table 2). Pasteur MLST scheme assigned a ST to 9 out of 14 (64.3%), and 7 out of 10 (70.0%) of the strains for ciprofloxacin and fosfomycin, respectively.

### 3.2. Identification of Point Mutations in Chromosomal or Plasmid Genes Involved in Ciprofloxacin or Fosfomycin Resistance

Additional analyses were performed on point mutations in the chromosomal and plasmid sequences to compare low-level resistance versus susceptible strains. For LLCR strains, 11 plasmid (*aac6*′*-1b-cr*, *qnrA*, *qnrB*, *qnrS*, *qnrC*, *qnrD*, *qnrE*, *qepA*, *oqxA*, *oqxB*, and *crpP*) and 8 chromosomal (*gyrA*, *gyrB*, *parC*, *parE*, *marR*, *acrR*, *sosX*, and *rpoB*) genes associated with quinolone resistance were analyzed. We found that most of the point mutations we detected had been previously described in the Comprehensive Antibiotic Resistance Database (CARD) as conferring quinolone resistance (Supplementary Table S2).

**Figure 1:**
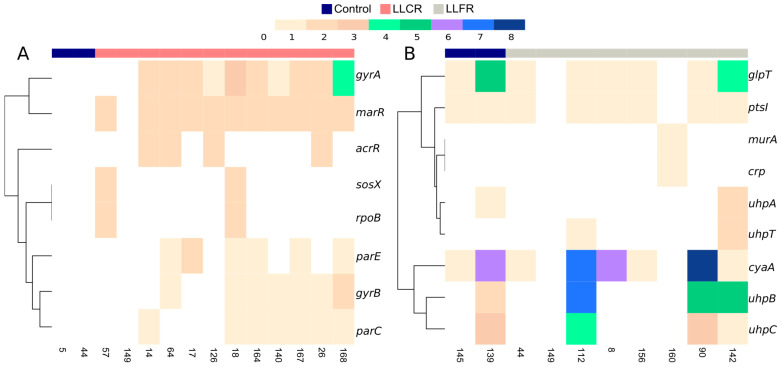
Heatmap of the accumulation of point mutations in the low-level ciprofloxacin resistance (LLCR) and low-level fosfomycin resistance (LLFR) strains. Abundance of point mutation in genes (axis Y) involved in (**A**) low-level quinolone resistance (LLCR, axis X) strains and (**B**) low-level fosfomycin resistance (LLFR, axis X) strains. Blue: Susceptible strains; Salmon: LLCR strains; Gray: LLFR strains; White: LLCR or LLFR strains with any point mutation in the studied genes involved in resistance. Range of colors from brown to blue: LLCR or LLFR strains with an accumulation from 1 to 8 point mutations in the studied genes involved in resistance.

**Figure 2:**
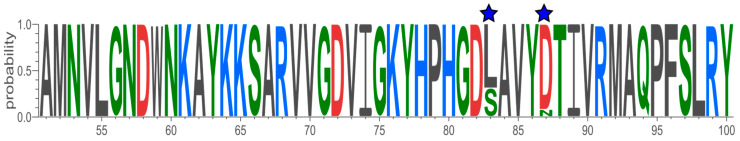
Logo of the amino acid sequence encoded by the *gyrA* gene in low-level ciprofloxacin resistance (LLCR) strains. Sequence conservation of amino acids encoding GyrA created from the aligned sequences of the 12 LLCR *E. coli* strains. Complete logo sequence is shown in Supplementary Figure S1. Letters depict the consensus amino acid of each position; Blue: Amino acids with side chain charge positively; Red: Amino acids with side chain charge negatively; Gray: No-polar amino acids; Green: Polar amino acids without changes; Blue star depict differences; The ggseqlogo library was used to create logo graph from the multiple sequence alignment.

Regarding chromosomal genes, 83% (10/12) of the strains presented a point mutation in the quinolone-resistance-determining region of DNA gyrase (*gyrA*) (Figure 1A), a target enzyme for quinolones [41]. Specifically, Ser83Leu and Asp87Leu point mutations were observed (Figure 2 and Supplementary Figure S1). Fifty percent (6/12) of the strains carried a non-previously described substitution mutation in *parC* and *parE* genes, encoding topoisomerase implied in bacterial replication [42], 33% also had a substitution in *gyrB* and *acrR*. While isolate 26 had a deletion in the *gyrB* gene, isolate 140 had a substitution plus deletion in the *gyrB* and *parC* genes. More than 91% (11/12) of the strains presented a point mutation in the *marR* gene, a repressor of the *marRAB* operon which is involved in the activation of both antibiotic resistance and oxidative stress genes [43]. Interestingly, all point mutations found in the already described genes involved in ciprofloxacin resistance were only present in LLCR strains and no point mutations were found in control strains. In addition, in plasmid mutations analysis, two strains (17%) carried a substitution in the *qnrS1* gene (Supplementary Table S2).

On the other hand, in LLFR strains, eight plasmid (*fosA3*, *fosA4*, *fosA5*, *fosB*, *fosX*, *fosC*, *fomA*, and *fomB*) and nine chromosomal (*murA*, *uhpA*, *uhpB*, *uhpC*, *uhpT*, *glpT*, *ptsl*, *cyaA*, and *crp*) genes were analyzed. Most of the point mutations found were previously described in the curated CARD database that confers resistance to fosfomycin (Supplementary Table S3).

**Figure 3:**
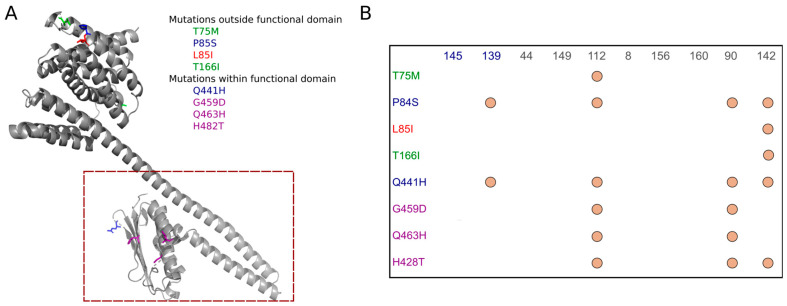
Presence of point mutation in UhpB protein. (**A**) Rendered structure and point mutation outside/within the functional domain and (**B**) abundance of point mutation in *uhpB* gene in the low-level fosfomycin resistance (LLFR) strains. In (**A**), green mutations: carried by LLFR strains outside of the functional domain (red square (IPR005467—Histidine kinase domain); blue mutations: carried by control strains outside or inside of the functional domain (red square); red mutation: protein due to this single amino acid variation is not stable; purple mutation: carried by LLFR strains outside of the functional domain (red square). In (**B**), orange dots: presence/absence of the mutation.

While none of the LLFR strains carried point mutations in the plasmidic genes tested, 75% (6/8) of the LLFR strains carried a point mutation in *cyaA*, *glpT*, and *ptsI* genes. Conversely to that observed in LLCR strains, these genes were mutated in one of the control susceptible strains tested (Figure 1B). However, the strains used as controls, since they present a susceptible phenotype in the antibiotics analyzed, are clinical strains isolated from UTI in KTR patients, and it is expected to find point mutations in them. In addition, 50% (4/8) of the strains carried substitutions in the *uhp* genes family, specifically 25% (2/8) of the LLFR strains carried a substitution affecting the coding region of hexose-phosphate transporter (UhpT) gene involved in the fosfomycin transportation into the bacteria [44]. Also, substitutions in the *uhp* genes family were observed in control susceptible strains, and only 3/9 (33%) chromosomic genes analyzed showed no mutations in control strains. Specifically, for the *uhpB* gene, some of the amino acidic substitutions carried by LLFR strains were found among positions from 311 to 499, the histidine kinase domain (Figure 3).

Finally, isolate 160 carried a point mutation in the *murA* and *crp* genes. Additionally, no mutations were observed in the analysis of plasmid mutations (Figure 1B and Supplementary Table S3).

Analysis for plasmid resistance genes found that all strains, both LLCR and LLFR, carried at least one plasmid (Supplementary Tables S2 and S3).

### 3.3. Identification of Unknown Resistance Chromosomal or Plasmid Genes and Virulence Factors in LLCR and LLFR Strains

Both the LLCR and LLFR strains showed at least one gene involved in Antimicrobial Resistance (AMR). Regarding the analysis of the presence of antimicrobial resistance genes in the selected strains exhibiting low-level resistance, 83% (10/12) of the LLCR strains carried mutations in *gyrA*, and 13% (1/8) of the LLFR strains carried *fosA*. This gene encodes metalloenzymes which hydrolyze fosfomycin [41]. Furthermore, most of these strains carried a wide variety of genes associated with resistance to aminoglycosides, β-lactams, sulfonamides, and tetracyclines (Table 3 and Table 4). However, no clear association was detected among the presence of these AMR genes and the strains’ MIC phenotype. Only two LLCR strains presented the aminoglycoside modifying enzymes (AMEs), *aac(3*′*)-IIa* (encoding tobramycin and gentamycin resistance) [45]. Similarly, AME was rarely present in LLFR strains, with only one strain presenting an *aac(3*′*)-Ib* involved in streptomycin resistance [46]. With respect to β-lactam resistance genes, both genes encoding class A and D β-lactamases were identified in all LLCR strains, especially, the *bla*_TEM-1_ gene, responsible for ampicillin, piperacillin, amoxicillin, and ticarcillin resistance [47]. Moreover, 62% (5/8) of the LLFR strains also carried the *bla*_TEM-1_ gene. However, 75% (9/12) and 37% (3/8) of the LLCR and LLFR strains, respectively, carried genes involved in folate pathway antagonists, conferring resistance to trimethoprim [48]. Furthermore, and regarding the sulfonamide resistance, genes encoding *sul1* and *sul2*, target enzyme dihydropteroate synthase [49], were found in 75% (9/12) and 50% (4/8) of LLCR and LLFR strains, respectively. Finally, 83% (10/12) of LLCR and 62% (5/8) of LLFR strains carried *tetA* or *tetB* genes, which encodes an efflux protein of the membrane that confers resistance to antibiotics of the tetracycline family, such as tetracycline, minocycline, and doxycycline [50].

**Figure 4:**
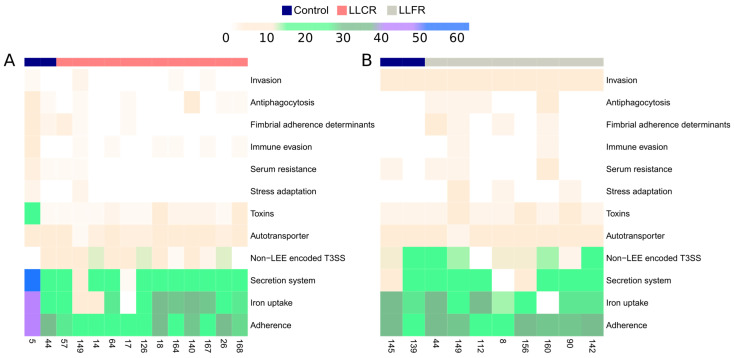
Heatmap of the abundance of virulence factor in the low-level ciprofloxacin resistance (LLCR) and low-level fosfomycin resistance (LLFR) strains. Presence of genes encoding virulence factor in: (**A**) low-level ciprofloxacin resistance (LLCR, axis X) strains, and (**B**) low-level fosfomycin resistance (LLFR, axis X) strains. Virulence factors were clustered in different classes (axis Y). Blue: Susceptible strains; Salmon: LLCR strains; Gray: LLFR strains; White: LLCR or LLFR strains with absence of genes encoding virulence factors. Range of colors from brown to blue: LLCR or LLFR strains carrying from 1 to 60 virulence factors. resistance.

In LLCR strains, several accessory genes belonged to virulence factor classes related to urinary tract infection were analyzed; specifically, genes involved in adherence, autotransporter, fimbriae, biofilm formation, immune evasion, secretion system including Type III secretion system (TTSS), toxin production, iron uptake, secretion system, and motility (Supplementary Table S4). Genes involved in adherence, autotransporter, and TTSS were identified in more than 50% of LLCR strains and were absent in susceptible-to-ciprofloxacin strains (Figure 4A). However, virulence genes encoded by LLCR strains could not be associated with the isolate phenotype (LLCR/susceptible), due to one of the ciprofloxacin-susceptible strains classified as control strains carried more than 40 virulence factors encoding genes compared to LLCR strains.

WGS and in silico analyses identified several chromosomal and plasmid-mediated genes associated with low-level resistance. Notably, two previously described point mutations in the *gyrA* gene (Ser83Leu and Asp87Leu) [51] were present in 83% of the LLCR strains. Additionally, the presence of *qnrS1* gene [52] in 17% of the LLCR strains suggests the potential for plasmid-mediated resistance contributing to the low-level resistance phenotype. These findings emphasize the multifaceted nature of quinolone resistance, where both chromosomal mutations and plasmid-encoded genes can interplay to confer resistance. Regarding LLFR, point mutation in *glpT* gene involved in fosfomycin resistance [53] was present in 75% of the studied strains. Mutations found in *cyaA* and *fosA* genes [54,55] were identified in 13% of the strains. Furthermore, 38% (3/8) of the analyzed strains carried a mutation in the *uhp* gene family (including *uhpA*, *uhpB*, *uhpC*, and *uhpT* genes). However, only 33% of the total chromosomal genes analyzed showed no mutations in the susceptible control strains, which may indicate that low-level resistance to fosfomycin may be due to a combination of point mutations in different genes [56] or, on the other hand, an accumulation of point mutations in a single gene [57]. Interestingly, this second hypothesis, also known as multi-step resistance evolution, was observed in *uhpB* gene, one of the LLFR-susceptible controls (139) and 38% LLFR strains amino acidic substitutions in the coding sequence. Specifically, control strain 139 carried two different point mutations, and the LLFR strains 112, 90, and 142 carried 7, 5, and 5, respectively, mutations at different positions in the gene sequence. Some of them were even found in the histidine kinase domain. However, no clear association was detected among the presence of these AMR genes and the strains’ MIC phenotype.

On the other hand, in LLFR strains, genes belonging to virulence factor classes related to urinary tract infection were found, including those involved in adherence, fimbriae, secretion system including Type III secretion system (TTSS), autotransporter, toxin production, antiphagocytosis, invasion and immune evasion (Supplementary Table S4). Genes involved in adherence were identified in more than 50% of LLFR strains and absent in susceptible-to-fosfomycin strains (Figure 4B). However, virulence genes encoded by LLFR strains could not be associated with the isolate phenotype (LLCR/susceptible).

## 4. Discussion

This study provides a comprehensive analysis of the genetic mechanisms underlying low-level resistance to ciprofloxacin and fosfomycin in uropathogenic *E. coli* strains, which is crucial for the development of new therapeutic alternatives to combat antibiotic resistance.

Remarkably, 50% of the LLFR strains exhibit resistance mechanisms related to the folate pathway. This high rate of resistance to sulfamethoxazole has been widely reported in cohorts of patients with urinary tract infections [58,59].

The main limitation of this study is the small number of strains analyzed, which makes it difficult to draw powerful conclusions. However, despite the limited sample size, all of these are clinical strains from a well-characterized cohort of KTR, and the incidence rates have already been assessed in these patients, with similar rates [20].

In conclusion, point mutations in the *gyrA* gene were the most prevalent found in the strains with low-level resistance to ciprofloxacin. In addition, all these strains carried a previously described mutation implicated in quinolone resistance. As for the strains with low-level fosfomycin resistance, they presented a low proportion of mutations in different genes such as *fosA*, *cyaA*, *uhpB*, and *uhpT* genes, which may imply novel or, even, an additive effect of the mutations in chromosomal and plasmid genes related to low-level fosfomycin resistance.

## Figures and Tables

**Table 1:** Multilocus sequence type and minimum inhibitory concentration of *Escherichia coli* strains with low resistance to ciprofloxacin.

Strains	MLST		MIC (µg/mL)
	Pasteur	Achtman	
5 *	ST509	ST625	<0.015 (S)
44 *	Unknown (nearest 74)	Unknown (nearest 12,985)	<0.015 (S)
57	ST831	ST1136	0.12
149	ST2	ST548	0.06
14	ST14	ST68	0.25
64	ST3	ST5021	0.12
17	Unknown (nearest 132)	ST48	0.12
126	ST14	ST68	0.50
18	Unknown (nearest 35)	Unknown (nearest 1439)	0.12
164	ST634	ST429	0.25
140	Unknown (nearest 24)	Unknown (nearest 889)	0.50
167	ST634	ST429	0.25
26	Unknown (nearest 14)	ST68	0.25
168	ST35	ST12	0.12

*: Clinical strains 5 and 44 were added as control susceptible (S) strains.

**Table 2:** Multi-locus sequence type and minimum inhibitory concentration of *Escherichia coli* strains with low resistance to fosfomycin.

Strains	MLST		MIC (µg/mL)
	Pasteur	Achtman	
**145** *	ST7	ST23	1 (S)
139 *	ST3	ST69	0.50 (S)
44	Unknown (nearest 74)	Unknown (nearest 12,985)	2
149	ST2	ST548	8
112	Unknown (nearest 966)	Unknown (nearest 4195)	2
8	ST83	ST93	2
156	ST471	ST410	2
160	ST2	ST10	2
90	ST32	ST127	2
142	Unknown (nearest 14)	ST68	2

*: Clinical strains 145 and 139 were added as control susceptible (S) strains.

**Table 3:** Analysis of the presence of antimicrobial resistance genes in the selected strains exhibiting low-level ciprofloxacin resistance.

Antibiotic Family Affected	Gene	Abundance in LLCR (%)	Specific Antimicrobials Affected
Aminoglycoside	*aac(3)-II*	25%	Tobramycin, sisomicin, apramycin, dibekacin, netilmicin
	*aph(3″)-I*	75%	Streptomycin
	*aph(6)-I*	75%	Streptomycin
	*aadA1*	42%	Streptomycin, spectinomycin
	*aadA5*	25%	Streptomycin, spectinomycin
Amphenicol	*catA1*	33%	Chloramphenicol
	*cmlA1*	8%	Chloramphenicol
	*floR*	33%	Florfenicol, chloramphenicol
β-lactam	*ampC*	8%	Cefoxitin, ampicillin, ampicillin/clavulanic acid, cefotaxime, ceftazidime
	*bla_CARB-2_*	8%	Ampicillin, piperacillin, amoxicillin
	*bla_EC_*	100%	Class C
	*bla_OXA-1_*	8%	Ampicillin, amoxicillin/clavulanic acid, piperacillin, amoxicillin, cefepime, piperacillin/tazobactam
	*bla_OXA-10_*	8%	Ampicillin, piperacillin/tazobactam, amoxicillin, piperacillin, aztreonam
	*bla_TEM-1A_*	83%	Ampicillin, cephalothin, piperacillin, amoxicillin, ticarcillin
	*bla_TEM-1B_*	83%	Ampicillin, cephalothin, piperacillin, amoxicillin, ticarcillin
Folate pathway antagonist	*dfrA1*	42%	Trimethoprim
	*dfrA8*	8%	Trimethoprim
	*dfrA14*	8%	Trimethoprim
	*dfrA17*	25%	Trimethoprim
	*dfrA36*	8%	Trimethoprim
	*sul1*	58%	Sulfamethoxazole
	*sul2*	67%	Sulfamethoxazole
	*sul3*	8%	Sulfamethoxazole
Fosfomycin	*cyaA*	8%	Fosfomycin
	*uhpT*	42%	Fosfomycin
Macrolide	*mph(A)*	42%	Erythromycin, telithromycin, azithromycin, spiramycin
Peroxide	*sitABCD*	58%	(hydrogen peroxide)
Quaternary ammonium compound	*qacE*	58%	Cetylpyridinium chloride, ethidium bromide, benzylkonium chloride, chlorhexidine
	*qacL*	8%	Cetylpyridinium chloride, ethidium bromide, benzylkonium chloride, chlorhexidine
Quinolone	*acr*	33%	Ciprofloxacin
	*gyrA*	83%	Ciprofloxacin and nalidixic acid
	*gyrB*	33%	Ciprofloxacin and nalidixic acid
	*parC*	50%	Ciprofloxacin and nalidixic acid
	*parE*	50%	Ciprofloxacin and nalidixic acid
	*qnrS1*	17%	Ciprofloxacin
Rifamycin	*ARR-2*	8%	Rifampicin
Tetracycline	*tet(A)*	58%	Doxycycline, tetracycline
	*tet(B)*	17%	Tetracycline, doxycycline, minocycline
Various	*erm(B)*	8%	Streptogramin B, lincosamide (clindamycin, lincomycin), and macrolide (erythromycin)

**Table 4:** Analysis of the presence of antimicrobial resistance genes in the selected strains exhibiting low-level fosfomycin resistance.

Antibiotic Family Affected	Gene	Abundance in LLFR (%)	Specific Antimicrobials Affected
Aminoglycoside	*aph(3″)-I*	50%	Streptomycin
	*aph(6)-I*	50%	Streptomycin
	*aadA1*	38%	Streptomycin, spectinomycin
Amphenicol	*catA1*	13%	Chloramphenicol
	*oqxB*	13%	Florfenicol, chloramphenicol
β-lactam	*bla_EC_*	88%	Class C
	*bla_MIR-6_*	13%	Ampicillin, amoxicillin/clavulanic acid, ticarcillin/clavulanic acid, cefoxitin, piperacillin, amoxicillin, piperacillin/tazobactam, ceftazidime, ticarcillin, cefotaxime, ampicillin/clavulanic acid
	*bla_TEM-1B_*	50%	Ampicillin, cephalothin, piperacillin, amoxicillin, ticarcillin
Folate pathway antagonist	*dfrA1*	42%	Trimethoprim
	*dfrA5*	8%	Trimethoprim
	*sul1*	25%	Sulfamethoxazole
	*sul2*	38%	Sulfamethoxazole
Fosfomycin	*cyaA*	13%	Fosfomycin
	*fosA*	13%	Fosfomycin
	*uhpT*	25%	Fosfomycin
Peroxide	*sitABCD*	38%	Hydrogen peroxide
Quaternary ammonium compound	*qacE*	25%	Cetylpyridinium chloride, ethidium bromide, benzylkonium chloride, chlorhexidine
Quinolone	*gyrA*	50%	Ciprofloxacin and nalidixic acid
	*parC*	38%	Ciprofloxacin
	*parE*	13%	Ciprofloxacin
Tetracycline	*tet(A)*	38%	Doxycycline, tetracycline
	*tet(B)*	25%	Tetracycline, doxycycline, minocycline
Various	*marR*	13%	Ampicillin, chloramphenicol, quinolone, rifampin, tetracycline

## Data Availability

The data presented in this study are available on request from the corresponding authors.

## Supplementary Materials

The following supporting information can be downloaded at: https://www.mdpi.com/article/10.3390/biom15020260/s1. Table S1: Demographics and characteristics of the kidney transplant recipients with urinary tract infection by *Escherichia coli* classified as low-level ciprofloxacin resistance (LLCR) or low-level fosfomycin resistance (LLFR); Table S2: Antimicrobial resistance genes encoded by plasmid found in LLCR and susceptible to ciprofloxacin *Escherichia coli* strains; Supplementary Table S3: Antimicrobial resistance genes encoded by plasmid found in LLFR and susceptible to fosfomycin *Escherichia coli* strains; Table S4: Virulence genes found in LLQR, LLFR, susceptible to ciprofloxacin or fosfomycin *Escherichia coli* strains; Figure S1: Sequence logo of the protein sequence coding *gyrA* gene in the 12 LLQR strains.
