# Supplementary material for: Detection of Possible Resistance Mechanisms in Uropathogenic Escherichia coli Strains Isolated from Kidney Transplant Recipients Based on Whole Genome Sequencing
Source: Biomolecules. 2025 Feb 11;15(2):260. doi: 10.3390/biom15020260 (PMC11853403; doi:10.3390/biom15020260)
Supplement: Supplementary file 1 [file biomolecules-15-00260-s001.zip › biomolecules-3442849-supplementary.pdf]

Supplementary Table 1. Demographics and characteristics of the kidney transplant recipients with urinary tract infection by *Escherichia coli* classified as low-level ciprofloxacin resistance (LLCR) or low-level fosfomycin resistance (LLFR).

**Variables**	**LLCR 12 episodes N (%)**	**LLFR 8 episodes N (%)**
Age (years; median [IQR])	58 (42-66)	50 (40-61)
Female patients	10 (83.3)	4 (50.0)
Charlson Comorbidity Index (median [IQR])	3 (1.25-3.0)	2 (2-4)
Months from transplantation (median [IQR])	30 (14.5-162)	12.5 (3.25-50.25)
< 2 months from transplantation	1 (8.3)	1 (12.5)
Previous kidney transplantation	2 (16.7)	2 (25.0)
Living donor	1 (8.3)	1 (12.5)
Induction therapy within 3 previous months:	6 (50.0)	5 (62.5)
- Thymoglobulin	3 (25.0)	2 (25.0)
- Basiliximab	3 (25.0)	3 (37.5)
- Daclizumab	0 (0.0)	0 (0.0)
Current immunosuppression:		
- Corticosteroids	10 (83.4)	9 (100.0)
- Tacrolimus	11 (91.7)	9 (100.0)
- MMF	8 (75.0)	9 (100.0)
- Cyclosporine	1 (8.3)	0 (0.0)
Acute rejection within the previous 6 months	1 (8.3)	0 (0.0)
Rejection treatment in the previous 6 months:		
- Corticosteroid's bolus	1 (8.3)	---
- Plasmapheresis	0 (0.0)	---
- Thymoglobulin	0 (0.0)	---
Creatinine (mg/dL; median [IQR])	1.36 (0.92-1.91)	1.41 (0.94-1.85)
Bacteriuria within the previous 6 months	4 (33.3)	6 (75.0)
Antibiotic use within the previous 3 months	3 (25.0)	6 (75.0)
- Quinolones*	1 (8.3)	1 (12.5)
- Amoxicillin-clavulanate	1 (8.3)	0 (0.0)
- Fosfomycin	0 (0.0)	2 (25.0)
- Cephalosporins**	1 (8.3)	0 (0.0)
- Others***	0 (0.0)	2 (25.0)
Cystitis	1 (8.3)	1 (12.5)
Asymptomatic bacteriuria	11 (91.7)	7 (87.5)
Urinary pH (median [IQR])	6.5 (6-6.9)	6 (5.6-6.4)
Urine pH <6	5 (41.7)	6 (75.0)
Baseline antibiotic resistance:		
- Cotrimoxazole	5 (58.3)	4 (44.4)
- Ciprofloxacin	0 (0.0)	1 (12.5)
- Amoxicillin-clavulanate	4 (33.3)	2 (25.0)
- Fosfomycin	2 (16.7)	0 (0.0)
- Cephalosporins****	0 (0.0)	2 (25.0)
Antibiotic therapy of the UTI episodes		
- Fosfomycin	8 (66.7)	7 (87.5)
- Ciprofloxacin	4 (33.3)	1 (12.5)

IQR: Interquartile range; MMF: Mycophenolate mofetil; ESBL: Extended spectrum beta-lactamases; * Quinolones: ciprofloxacin or levofloxacin; ** Cephalosporins: cefixime or cefuroxime; *** Others: Ertapenem; **** Cephalosporins: cefuroxime, cefotaxime, ceftazidime, cefixime or cefepime.

Supplementary Table S2. Antimicrobial resistance genes encoded by plasmid found in LLCR and susceptible to ciprofloxacin *Escherichia coli* strains.

**Plasmids**	**5**	**44**	**57**	**149**	**14**	**64**	**17**	**126**	**18**	**164**	**140**	**167**	**26**	**168**	**Abundance (%)**
IncFIB(AP001918)	-	+	+	-	-	+	-	-	-	+	+	+	+	+	58%
Col156	-	-	-	-	+	-	-	-	-	-	+	+	+	+	42%
IncI1-I(α)	-	-	-	-	+	-	-	+	+	-	+	-	+	-	42%
IncFIC(FII)	-	+	-	-	-	-	-	-	-	+	+	+	+	-	33%
IncFII(pRSB107)	-	-	+	+	-	+	-	-	-	-	-	-	-	-	25%
IncB/O/K/Z	-	-	+	-	-	-	-	-	+	-	-	-	-	-	17%
IncFIA	-	-	-	-	-	-	-	-	+	-	+	-	-	-	17%
IncI2	-	-	+	-	-	-	-	-	-	-	-	-	-	-	8%
Inc1-I(γ)	-	-	-	+	-	-	-	-	-	-	-	-	-	-	8%
IncR	-	-	-	+	-	-	-	-	-	-	-	-	-	-	8%
IncFII(29)	-	-	-	-	-	-	-	-	-	-	-	-	-	+	8%
IncHI2	-	-	-	-	-	-	+	-	-	-	-	-	-	-	8%
IncHI2A	-	-	-	-	-	-	+	-	-	-	-	-	-	-	8%
p0111	-	-	-	-	-	-	+	-	-	-	-	-	-	-	8%

+: Presence; -: Absence.

Supplementary Table S3. Antimicrobial resistance genes encoded by plasmid found in LLFR and susceptible to fosfomycin *Escherichia coli* strains.

Plasmids	145	139	44	149	112	8	156	160	90	142	Abundance (%)
IncFIB(AP001918)	+	-	+	-	+	+	+	-	-	-	50%
IncFIB(pECLA)	-	-	-	-	-	-	-	-	-	+	13%
IncFIC(FII)	-	-	+	-	-	-	-	-	-	-	13%
IncFII	+	-	+	-	-	-	+	-	-	-	25%
IncQ1	-	-	-	-	-	-	-	-	-	-	0%
IncFIB(pB171)	-	-	-	-	-	-	-	-	+	-	13%
IncFII(pRSB107)	-	-	-	+	-	+	-	-	+	-	38%
IncI1-I(α)	-	-	-	-	-	-	+	+	-	-	25%
Inc1-I(γ)	-	-	-	+	-	-	-	-	-	-	13%
IncR	-	-	-	+	-	-	-	-	-	-	13%
Col156	-	-	-	-	-	+	+	-	+	-	38%
IncFII(29)	-	-	-	-	-	-	-	+	-	-	13%
IncFII(pECLA)	-	-	-	-	-	-	-	-	-	+	13%
IncFIA	-	-	-	-	-	+	+	-	+	-	38%
IncFII(pAMA1167-NDM-5)	-	-	-	-	-	+	-	-	-	-	13%

+: Presence; -: Absence.

Supplementary Table S4. Virulence genes found in LLQR, LLFR, susceptible to ciprofloxacin or fosfomycin *Escherichia coli* strains.

Virulence factor class	Virulence factors	Genes	Lack in reference strain and presence (%) in LLQR strains	Presence in reference isolates (5 and/or 44) and lack in LLQR strains	Virulence factors	Genes	Lack in reference strain and presence (%) in LLFR strains	Presence in reference isolates (139 and/or 145) and lack in LLQR strains
Adherence	CFA / I fimbriae	*cfaA*			CFA/I fimbriae	*cfaA*		
		cfaB				*cfaB*		
		cfaC				*cfaC*		
		cfaD/cfaE	8%			*cfaD/cfaE*		Isolate 145
	Curli fibers	*cgsD*		Isolate 5	Curli fibers	*cgsD*		
		cgsF		Isolate 5		*cgsF*		
		cgsG		Isolate 5		*cgsG*		
		csgA		Isolate 5		*csgA*		
		csgB		Isolate 5		*csgB*		
		csgC		Isolate 5		*csgC*		
	ECP (E. coli *common pilus)*	*ecpA*			ECP *(E. coli common pilus)*	*ecpA*		
		ecpB				*ecpB*		
		ecpC				*ecpC*		
		ecpD				*ecpD*		
		ecpE				*ecpE*		
		ecpR				*ecpR*		
	ELF (*E. coli* laminin-binding fimbriae)	*elfA*	58%		ELF (E. coli laminin-binding fimbriae)	*elfA*		
		elfC				*elfC*		
		elfD				*elfD*		
		elfG				*elfG*		
	EaeH	*eaeH*			EaeH	*eaeH*		
	F1C fimbriae	*focC*			F1C fimbriae	*focC*	13%	
		focD				*focD*	13%	
		focF				*focF*	13%	

		focG				*focG*	13%	
		focH				*focH*	13%	
		focI				*focI*	13%	
	HCP (Haemorrhagic *E. coli* pilus)	*hcpA*			HCP (Haemorrhagic E. coli pilus)	*hcpA*		
		hcpB				*hcpB*		
		hcpC				*hcpC*		
	P fimbriae	*papA*	25%		P fimbriae	*papA*	0%	
		papB	8%			*papB*		
		papC				*papC*	38%	
		papD				*papD*	38%	
		papE	25%			*papE*		
		papF	8%			*papF*		
		papG				*papG*	13%	
		papH				*papH*	13%	
		papI				*papI*	25%	
		papJ	8%			*papJ*		
		papK				*papK*	13%	
	S fimbriae	*sfaB*		Isolate 5	S fimbriae	*sfaB*		
		sfaC		Both		*sfaC*	13%	
		sfaD		Isolate 5		*sfaD*		
		sfaF		Isolate 5		*sfaF*		
		sfaG		Isolate 5		*sfaG*		
	Type I fimbriae	*fimA*			Type I fimbriae	*fimA*	0%	
		fimB				*fimB*		
		fimC				*fimC*		
		fimD				*fimD*		
		fimE				*fimE*		

		fimF				*fimF*		
		fimG				*fimG*		
		fimH				*fimH*		
		fimI				*fimI*		
	Type IV pili (*Yersinia*)	*pilQ*	50%		Type IV pili (*Yersinia*)	*pilQ*	50%	
		pilR	50%			*pilR*	50%	
		pilS	33%			*piliS*		
		pilV	33%			*pilV*	13%	
		pilW				*pilW*	38%	
	Hsp60 (*Legionella*)	*htpB*		Isolate 5	Hsp60 (*Legionella*)	*htpB*		
	Lateral flagella (*Aeromonas*)	*flgC*	8%		Lateral flagella (*Aeromonas*)	*flgC*	8%	
		lfhA		Isolate 5		*lfhA*		
	Polar flagella (*Aeromonas*)	*flmH*		Isolate 5	Polar flagella (*Aeromonas*)	*flmH*		
		nueA		Isolate 5		*nueA*		
	Streptococcal plasmin receptor/APDH (*Streptococcus*)	*plr/gapA*		Isolate 5	Streptococcal plasmin receptor/APDH (*Streptococcus*)	*plr/gapA*		
	Type IV pili (*Neisseria*)	*pilT*		Isolate 5	Type IV pili (*Neisseria*)	*pilT*		
	LPS O-antigen (*P. aeruginosa*)		8%		LPS O-antigen (*Pseudomonas*)		0%	
Autotransporter	AatA	*aatA*	50%		AatA	*aatA*	25%	
	Antigen 43	*agn43*			Antigen 43	*agn43*		
	Cah	*cah*	17%		Cah	*cah*	25%	
	Contact-dependent inhibition CDI system	*cdiA*	17%		Contact-dependent inhibition CDI system			
		cdiB						

	EhaA	*ehaA*	17%		EhaA	*ehaA*	0%	
	EhaB	*ehaB*			EhaB	*ehaB*		
	Enter aggregative immunoglobulin repeat protein	*air/eaeX*	17%		Enteroaggregative immunoglobulin repeat protein	*air/eaeX*		Ambos
	EspC	*espC*	17%		EspC	*espC*	13%	
	EspI	*espI*	17%		EspI	*espI*	13%	
	EspP	*espP*		Isolate 44	EspP	*espP*	13%	
	Sat	*sat*	8%		Sat	*sat*	0%	
	Temperature-sensitive hemagglutinin	*tsh*			Temperature-sensitive hemagglutinin	*tsh*	13%	
	UpaG adhesin	*upaG/ehaG*			UpaG adhesin	*upaG/ehaG*		
	UpaH	*upaH*		Isolate 5	UpaH	*upaH*		
	Vacuolating autotransporter gene	*vat*			Vacuolating autotransporter gene	*vat*	38%	
Invasion	Ibes (Invasion of brain endothelial cells)	*ibeA*		Isolate 5	Invasion of brain endothelial cells (Ibes)	*ibeA*		
		ibeB				*ibeB*		
		ibeC				*ibeC*		
	Tia/Hek	*tia*			Tia/Hek	*tia*	25%	
	Flagella (*Burkholderia*)	*cheB*		Isolate 5	Flagella (*Burkholderia*)	*cheB*		
		cheR		Isolate 5		*cheR*		
		cheW		Isolate 5		*cheW*		
		cheY		Isolate 5		*cheY*		
		cheZ		Isolate 5		*cheZ*		
		motA		Isolate 5		*motA*		
Iron uptake		*iucA*				*iucA*		

	Aerobactin siderophore	*iucB*			Aerobactin siderophore	*iucB*		
		iucC				*iucC*		
		iucD				*iucD*		
		iutA				*iutA*		
	Heme uptake	*chuA*			Heme uptake	*chuA*		
		chuS				*chuS*		
		chuT				*chuT*		
		chuU				*chuU*		
		chuW				*chuW*		
		chuX				*chuX*		
		chuY				*chuY*		
	Iron-regulated element	*ireA*	25%		Iron-regulated element	*ireA*		
	Iron/manganese transport	*sitA*			Iron/manganese transport	*sitA*		
		sitB				*sitB*		
		sitC				*sitC*		
		sitD				*sitD*		
	Salmochelin siderophore	*iroB*			Salmochelin siderophore	*iroB*		
		iroC				*iroC*		
		iroD				*iroD*		
		iroE				*iroE*		
		iroN				*iroN*		
	Yersiniabactin siderophore	*fyuA*			Yersiniabactin siderophore	*fyuA*		
		irp1				*irp1*		
		irp2				*irp2*		
		ybtA				*ybtA*		

		ybtE				*ybtE*		
		ybtP				*ybtP*		
		ybtQ				*ybtQ*		
		ybtS				*ybtS*		
		ybtT				*ybtT*		
		ybtU				*ybtU*		
		ybtX				*ybtX*		
	Ccm locus (Cytochrome c maturation) (*Legionella*)	*ccmF*		Isolate 5	Ccm locus (Cytochrome c maturation) (*Legionella*)	*ccmF*		
	Ent siderophore (*Klebsiella*)			Isolate 5	Ent siderophore (*Klebsiella*)			
				Isolate 5				
	Enterobactin synthesis (*Shigella*)	*entA*		Isolate 5	Enterobactin synthesis (*Shigella*)	*entA*		
		entB		Isolate 5		*entB*		
		entC		Isolate 5		*entC*		
		entD		Isolate 5		*entD*		
		entE		Isolate 5		*entE*		
		entF		Isolate 5		*entF*		
	Enterobactin transport (*Shigella*)	*fepB*		Isolate 5	Enterobactin transport (*Shigella*)	*fepB*		
		fepC		Isolate 5		*fepC*		
		fepD		Isolate 5		*fepD*		
		fepG		Isolate 5		*fepG*		
	Heme biosynthesis (*Haemophilus*)	*hemC*		Isolate 5	Heme biosynthesis (*Haemophilus*)	*hemC*		
		hemE		Isolate 5		*hemE*		
		hemH		Isolate 5		*hemH*		
		hemL		Isolate 5		*hemL*		

		hemN		Isolate 5		*hemN*		
	Heme transport (*Shigella*)	*shuV*		Isolate 5	Heme transport (*Shigella*)	*shuV*		
Non-LEE encoded TTSS effectors	EspL1	*espL1*	92%		EspL1	*espL1*		
	EspL4	*espL4*			EspL4	*espL4*		
	EspR1	*espR1*			EspR1	*espR1*		
	EspR4	*espR4*	33%		EspR4	*espR4*		
	EspV	*espV*			EspV	*espV*	13%	
	EspX1	*espX1*			EspX1	*espX1*		
	EspX2	*espX2*	25%		EspX2	*espX2*	13%	
	EspX4	*espX4*			EspX4	*espX4*		
	EspX5	*espX5*			EspX5	*espX5*		
	EspX6	*espX6*	25%		EspX6	*espX6*	13%	
	EspY1	*espY1*	25%		EspY1	*espY1*		
	EspY2	*espY2*	25%		EspY2	*espY2*		
	EspY3	*espY3*	25%		EspY3	*espY3*		
	EspY4	*espY4*	25%		EspY4	*espY4*		
	NleA	*nleA*			NleA	*nleA*	13%	
	NleB2-1	*nleB2-1*			NleB2-1	*nleB2-1*	13%	
	NleG7	*nleG7*			NleG7	*nleG7*	13%	
	NleH1-1	*nleH1-1*			NleH1-1	*nleH1-1*	13%	
Regulation	Alternative sigma factor RpoS (*Legionella*)	*rpoS*		Isolate 5	Alternative sigma factor RpoS (*Legionella*)	*rpoS*		
	Carbon storage regulator A (*Legionella*)	*csrA*		Isolate 5	Carbon storage regulator A (*Legionella*)	*csrA*		
	GacS/GacA two-component system (*Pseudomonas*)	*gacA*		Isolate 5	GacS/GacA two-component system (*Pseudomonas*)	*gacA*		

	PhoPQ (*Salmonella*)	*phoP*		Isolate 5	PhoPQ (*Salmonella*)	*phoP*		
		phoQ		Isolate 5		*phoQ*		
	RcsAB (*Klebsiella*)	*rcsA*		Isolate 5	RcsAB (*Klebsiella*)	*rcsA*		
Secretion system	ACE T6SS	Undetermined			ACE T6SS	Undetermined		
		Undetermined				Undetermined	13%	
		aec14				*aec14*	13%	
		aec15				*aec15*		
		aec16				*aec16*		
		aec17				*aec17*		
		aec18				*aec18*		
		aec19				*aec19*		
		aec22				*aec22*		
		aec23				*aec23*		
		aec24				*aec24*		
		aec25				*aec25*		
		aec26				*aec26*		
		aec27/clpV				*aec27/clpV*		
		aec28				*aec28*		
		aec29				*aec29*		
		aec30				*aec30*		
		aec31				*aec31*		
		aec32				*aec32*		
		aec7	8%			*aec7*		
		aec8	17%			*aec8*	0%	

	icm/dot type IVB locus (*Yersinia*)		8%		icm/dot type IVB locus (*Yersinia*)		25%	
			33%		Flagella (cluster I) (*Yersinia*)	*fliC*	13%	
	EPS type II secretion system (*Vibrio*)	*epsE*		Isolate 5	EPS type II secretion system (*Vibrio*)	*epsE*		
	Flagella (cluster I) (*Yersinia*)	*flgB*		Isolate 5	Flagella (cluster I) (*Yersinia*)	*flgB*		
		flgC		Isolate 5		*flgC*		
		flgD		Isolate 5		*flgD*		
		flgE		Isolate 5		*flgE*		
		flgF		Isolate 5		*flgF*		
		flgG				*flgG*		
		flgH				*flgH*		
		flgI		Isolate 5		*flgI*		
		flgJ		Isolate 5		*flgJ*		
		flgK		Isolate 5		*flgK*		
		flgL		Isolate 5		*flgL*		
		flgM		Isolate 5		*flgM*		
		flhA		Isolate 5		*flhA*		
		flhB		Isolate 5		*flhB*		
		flhC		Isolate 5		*flhC*		
		flhD		Isolate 5		*flhD*		
		fliA		Isolate 5		*fliA*		
		fliC	8%			*fliC*		
		fliE		Isolate 5		*fliE*		
		fliF		Isolate 5		*fliF*		
		fliG		Isolate 5		*fliG*		
		fliH		Isolate 5		*fliH*		

		fliI	Isolate 5		*fliI*	
		fliJ	Isolate 5		*fliJ*	
		fliL	Isolate 5		*fliL*	
		fliM	Isolate 5		*fliM*	
		fliN	Isolate 5		*fliN*	
		fliP	Isolate 5		*fliP*	
		fliQ	Isolate 5		*fliQ*	
		fliR	Isolate 5		*fliR*	
		fliS	Isolate 5		*fliS*	
		fliZ	Isolate 5		*fliZ*	
	Mxi-Spa TTSS effectors controlled by MxiE (*Shigella*)	*ipaH*	Isolate 5	Mxi-Spa TTSS effectors controlled by MxiE (*Shigella*)	*ipaH*	
	T2SS (Type II secretion system) (*Shigella*)	*gspC*	Isolate 5	T2SS (Type II secretion system) (*Shigella*)	*gspC*	
		gspD	Isolate 5		*gspD*	
		gspE	Isolate 5		*gspE*	
		gspF	Isolate 5		*gspF*	
		gspG	Isolate 5		*gspG*	
		gspH	Isolate 5		*gspH*	
		gspI	Isolate 5		*gspI*	
		gspJ	Isolate 5		*gspJ*	
		gspK	Isolate 5		*gspK*	
		gspL	Isolate 5		*gspL*	
		gspM	Isolate 5		*gspM*	
	T2SS (*Aeromonas*)	*exeD*	Isolate 5	T2SS (*Aeromonas*)	*exeD*	
		exeG	Isolate 5		*exeG*	
	T4SS effectors (*Coxiella*)		Isolate 5	T4SS effectors (*Coxiella*)		
			Isolate 5			

	T6SS-II (*Klebsiella*)			Isolate 5	T6SS-II (*Klebsiella*)			
	T6SS (*Aeromonas*)			Isolate 5	T6SS (*Aeromonas*)			
	Lvh (Legionella vir homologs) type IVA secretion system (*Legionella*)	*traG*	8%		Lvh (Legionella vir homologs) type IVA secretion system (*Legionella*)	*traG*	13%	
		trbE	8%			*trbE*	13%	
		virB	8%			*virB*	13%	
Toxin	Alpha-hemolysin	*hlyA*			Alpha-hemolysin	*hlyA*		
		hlyB				*hlyB*		
		hlyC	17%			*hlyC*		
		hlyD				*hlyD*		
	Colicin-like Usp	*usp*			Colicin-like Usp	*usp*	13%	
	Cytotoxic necrotizing factor 1	*cnf1*			Cytotoxic necrotizing factor 1	*cnf1*	13%	
	Enterotoxin SenB/TieB	*senB*	33%		Enterotoxin SenB/TieB	*senB*	25%	
	Hemolysin/cytolysin A	*hlyE/clyA*			Hemolysin/cytolysin A	*hlyE/clyA*		
	Colibactin (*Klebsiella*)	*clbA*		Isolate 5	Colibactin (*Klebsiella*)	*clbA*		
		clbB		Isolate 5		*clbB*		
		clbC		Isolate 5		*clbC*		
		clbD		Isolate 5		*clbD*		
		clbE		Isolate 5		*clbE*		
		clbF		Isolate 5		*clbF*		
		clbG		Isolate 5		*clbG*		
		clbH		Isolate 5		*clbH*		
		clbI		Isolate 5		*clbI*		
		clbJ		Isolate 5		*clbJ*		

		clbK		Isolate 5		*clbK*		
		clbL		Isolate 5		*clbL*		
		clbM		Isolate 5		*clbM*		
		clbN		Isolate 5		*clbN*		
		clbO		Isolate 5		*clbO*		
		clbP		Isolate 5		*clbP*		
		clbQ		Isolate 5		*clbQ*		
	Heat-stable cytotonic enterotoxin	*ast*		Isolate 5	Heat-stable cytotonic enterotoxin	*ast*		
	Hemolysin HlyA (*Aeromonas*)	*hlyA*		Isolate 5	Hemolysin HlyA (*Aeromonas*)	*hlyA*		
	Hemolysin III (*Aeromonas*)			Isolate 5	Hemolysin III (*Aeromonas*)			
	Phytotoxin phaseolotoxin (*Pseudomonas*)	*cysC1*		Isolate 5	Phytotoxin phaseolotoxin (*Pseudomonas*)	*cysC1*		
Amino acid and purine metabolism	Glutamine synthesis (*Mycobacterium*)	*glnA1*		Isolate 5	Glutamine synthesis (*Mycobacterium*)	*glnA1*		
Anaerobic respiration	Nitrate reductase (*Mycobacterium*)	*narH*		Isolate 5	Nitrate reductase (*Mycobacterium*)	*narH*		
Antiphagocytosis	Alginate regulation (*Pseudomonas*)	*algU*		Isolate 5	Alginate regulation (*Pseudomonas*)	*algU*		
	Capsular polysaccharide (*Vibrio*)	*rmlB*		Isolate 5	Capsular polysaccharide (*Vibrio*)	*rmIA*	13%	
		wbjD/wecB				*wbjD/wecB*	25%	
		wecC		Isolate 5		*wecC*		
	Capsule (*Klebsiella*)	*galF*		Isolate 5	Capsule (*Klebsiella*)		13%	

		wcaI		Isolate 5		*uge*	0%	
		probable wbaZ				*probable wbaZ*	0%	
		wbaP				*wzc*	13%	
		wzc				*wzi*	13%	
Biofilm formation	PNAG (Polysaccharide poly-N-acetylglucosamine) (*Acinetobacter*)	*pgaC*		Isolate 5	PNAG (Polysaccharide poly-N-acetylglucosamine) (*Acinetobacter*)	*pgaC*		
Cell surface components	Trehalose-recycling ABC transporter (*Mycobacterium*)	*sugC*		Isolate 5	Trehalose-recycling ABC transporter (*Mycobacterium*)	*sugC*		
Colonization and Immune evasion	Capsule biosynthesis and transport (*Campylobacter*)	*glf*		Isolate 5	Capsule biosynthesis and transport (*Campylobacter*)	*glf*		
		kpsT		Isolate 5		*kpsT*		
Efflux pump	AcrAB (*Klebsiella*)			Isolate 5	AcrAB (*Klebsiella*)			
				Isolate 5				
		acrB		Isolate 5		*acrB*		
	FarAB (*Neisseria*)	*farB*		Isolate 5	FarAB (*Neisseria*)	*farB*		
Endotoxin	LOS (*Haemophilus*)	*gmhA/lpcA*		Isolate 5	LOS (*Haemophilus*)	*gmhA/lpcA*		
		htrB		Isolate 5		*htrB*		
		kdsA		Isolate 5		*kdsA*		
		kdtA		Isolate 5		*kdtA*		
		kpsF		Isolate 5		*kpsF*		
		lpxA		Isolate 5		*lpxA*		
		lpxB		Isolate 5		*lpxB*		
		lpxC		Isolate 5		*lpxC*		

		lpxD		Isolate 5		*lpxD*		
		lpxK		Isolate 5		*lpxK*		
		msbA		Isolate 5		*msbA*		
		opsX/rfaC		Isolate 5		*opsX/rfaC*		
		orfM		Isolate 5		*orfM*		
		rfaD		Isolate 5		*rfaD*		
		rfaE		Isolate 5		*rfaE*		
		rfaF		Isolate 5		*rfaF*		
		wecA		Isolate 5		*wecA*		
Enzyme	Streptococcal enolase (*Streptococcus*)	*eno*		Isolate 5	Streptococcal enolase (*Streptococcus*)	*eno*		
Fimbrial adherence determinants	Lpf (*Salmonella*)	*lpfB*	8%		Lpf (*Salmonella*)	*lpfB*		
		lpfC	8%			*lpfC*		
		lpfE	8%			*lpfE*		
	Sta (*Salmonella*)	*staB*		Isolate 5	Sta (*Salmonella*)	*staB*		
		staC		Isolate 5		*staC*		
	Stc (*Salmonella*)	*stcB*		Isolate 5	Stc (*Salmonella*)	*stcB*		
		stcC		Isolate 5		*stcC*		
		stcD		Isolate 5		*stcD*		
	Stf (*Salmonella*)	*stfC*		Isolate 5	*Stf* (*Salmonella*)	*stfD*	13%	
		stfD				*stfE*	13%	
		stfE		Isolate 5				
		stfF		Isolate 5		*stjC*	38%	
	Stj (*Salmonella*)	*stjC*			*Stj* (*Salmonella*)	*stjC*		
Immune evasion		*galE*		Isolate 5	Exopolysaccharide (*Haemophilus*)	*galE*	13%	

	Exopolysaccharide (*Haemophilus*)	*galU*		Isolate 5				
	Exopolysaccharide (*Haemophilus*)	*mrsA/glmM*		Isolate 5				
	Exopolysaccharide (*Haemophilus*)	*pgi*		Isolate 5				
	LPS (*Brucella*)	*acpXL*		Isolate 5	LPS (*Brucella*)	*acpXL*		
	Capsule (*Acinetobacter*)		42%		Capsule (*Acinetobacter*)		13%	
Lipid and fatty acid metabolism	Isocitrate lyase (*Mycobacterium*)	*icl*		Isolate 5	Isocitrate lyase (*Mycobacterium*)	*icl*		
Lipid and fatty acid metabolism	Pantothenate synthesis (*Mycobacterium*)	*panD*		Isolate 5	Pantothenate synthesis (*Mycobacterium*)	*panD*		
Magnesium uptake	Mg^{2+} transport (*Salmonella*)	*mgtB*		Isolate 5	Mg^{2+} transport (*Salmonella*)	*mgtB*		
Motility	Flagella (*Bordetella*)	*motB*		Isolate 5	Flagella (*Bordetella*)	*motB*		
Nonfimbrial adherence determinants	SinH (*Salmonella*)	*sinH*		Isolate 5	SinH (*Salmonella*)	*sinH*		
Nutritional factor	Allantoin utilization (*Klebsiella*)			Isolate 5	Allantoin utilization (*Klebsiella*)			
Nutritional factor	Allantoin utilization (*Klebsiella*)			Isolate 5				
Nutritional factor	Allantoin utilization (*Klebsiella*)			Isolate 5				
Nutritional factor	Allantoin utilization (*Klebsiella*)			Isolate 5				
Nutritional factor	Allantoin utilization (*Klebsiella*)			Isolate 5				
Nutritional factor	Allantoin utilization (*Klebsiella*)			Isolate 5				
Nutritional virulence	Biotin metabolism (*Francisella*)	*bioB*		Isolate 5	Biotin metabolism (*Francisella*)	*bioB*		
Nutritional virulence	Cysteine acquisition (*Francisella*)			Isolate 5	Cysteine acquisition (*Francisella*)			

	Pyrimidine biosynthesis (*Francisella*)			Isolate 5	Pyrimidine biosynthesis (*Francisella*)			
Others	MsbB2 (*Shigella*)	*msbB2*		Isolate 5	O-antigen (*Yersinia*)		13%	
	O-antigen (*Yersinia*)					*galE*	13%	
Protease	IcsP (*SopA*)(*Shigella*)	*icsP/sopA*		Isolate 5		*cpsB*	13%	
Quorum sensing	Autoinducer-2 (*Vibrio*)	*luxS*		Isolate 5		*wbyL*	13%	
Serum resistance	LPS *rfb* locus (*Klebsiella*)	*rmlA*		Isolate 5	LPS *rfb* locus (*Klebsiella*)	*rmlD*	13%	
Serum resistance and immune evastion	Capsule (*Francisella*)			Isolate 5	Capsule (*Francisella*)			
	LPS (*Francisella*)			Isolate 5	LPS (*Francisella*)			
Stress adaptation	Catalase-peroxidase (*Mycobacterium*)	*katG*		Isolate 5	Catalase-peroxidase (*Mycobacterium*)	*katG*		
	SodCI (*Salmonella*)	*sodCI*		Isolate 5	*SodCI* (*Salmonella*)	*sodCI*		
Motility	Flagella (*Bordetella*)	*flaA*	8%		Flagella (*Bordetella*)	*flaA*		

Supplementary Figure S1. Sequence logo of the protein sequence coding *gyrA* gene in the LLQR strains.

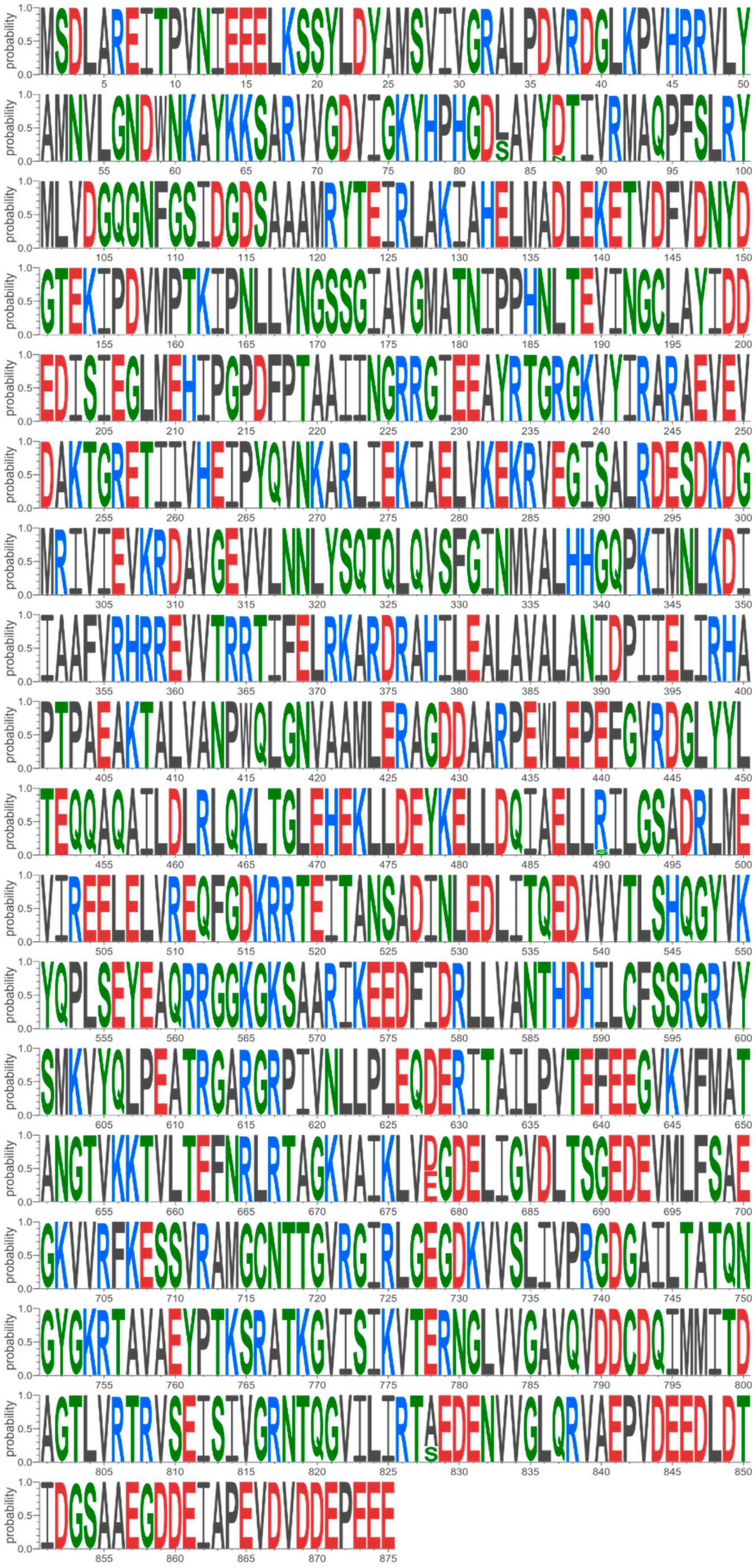

Graphical representation of the sequence conservation of amino acids coding GyrA created from the aligned sequences of the 12 LLQR *E. coli* strains. Letters depicts the consensus amino acid of each position; Blue: Amino acids with side chain charge positively; Red: Amino acids with side chain charge negatively; Grey: No-polar amino acids; Green: Polar amino acids without changes; The ggseqlogo library was used to create logo graph from the multiple sequence alignment.
